# Occurrence and Genetic Diversity of *Cryptosporidium* spp. and *Giardia intestinalis* from Yaks (*Bos grunniens*) in Ganzi, Sichuan Province, China

**DOI:** 10.3390/microorganisms13061261

**Published:** 2025-05-29

**Authors:** Yingying Fan, Guirong Hu, Danjiao Yang, Xinrui Hou, Mingyi Zhang, Yufeng Niu, Zijie Wang, Xin Yang

**Affiliations:** College of Veterinary Medicine, Northwest A&F University, Yangling 712100, China; yingyingfan@nwafu.edu.cn (Y.F.); hgr18637979425@163.com (G.H.); kangyang2468@126.com (D.Y.); hxrdyx2001@163.com (X.H.); 2019010830@nwafu.edu.cn (M.Z.); smtismyxn@outlook.com (Y.N.); wangzijie@nwafu.edu.cn (Z.W.)

**Keywords:** *Cryptosporidium*, *Giardia intestinalis*, yak, Ganzi

## Abstract

*Cryptosporidium* spp. and *Giardia intestinalis* are important zoonotic protozoa that are closely related to diarrhea and cause considerable economic losses in the livestock breeding industry. Ganzi is one of the main production areas for yaks in China, but there have been few reports on the occurrence of *Cryptosporidium* spp. and *G. intestinalis* in yaks. This study used PCR-based sequencing techniques to survey the prevalence and species/genotypes of *Cryptosporidium* spp. and *G. intestinalis* in faecal samples from 223 yaks in Ganzi, Sichuan Province. The positive rate of *Cryptosporidium* spp. was 7.2% (16/223), with the highest positive rate of yaks found in Yajiang (37%, 10/27), which was significantly higher than that in Litang (3.1%, 4/130) and Seda (3%, 2/66). The positive rate of *Cryptosporidium* spp. in young animals aged <6 months (20.5%, 8/39) was significantly higher than that in older animals aged 12–23 months (0; 0/43) and >24 months (3.3%, 3/90). Three *Cryptosporidium* species were found by sequence analysis of *18S* rRNA locus, namely *C. andersoni*, *C. ryanae*, and *C. bovis*. The total positive rate of *G. intestinalis* was 15.7% (35/223), with significant differences identified between Yajiang (40.7%, 11/27), Litang (17.7%, 23/130), and Seda (1.5%, 1/66). One genotype (assemblage E) was found by analyzing the sequence of *gdh*, *bg*, and *tpi* loci. Meanwhile, co-infection of *Cryptosporidium* spp. and *Giardia intestinalis* was identified in five samples. The present study explores the infection of *Cryptosporidium* spp. and *G. intestinalis* from yaks in Ganzi, aiming to enrich our understanding of the occurrence of these protozoa in livestock.

## 1. Introduction

*Cryptosporidium* spp. and *Giardia intestinalis* are common zoonotic protozoa leading to diarrhea in quite a few animals and humans [1,2]. These two protozoa can be transmitted by foodborne and waterborne pathways or direct/indirect contact with infected hosts, threatening the health of humans and the development of the breeding industry [1,3,4]. Oocysts of the two pathogens can be excreted in faeces from the hosts, and then contaminate food and water. Humans and animals are usually infected by ingesting polluted food and water or coming into contact with faeces containing oocysts [1,3,4]. As important opportunistic pathogens, *Cryptosporidium* spp. and *G. intestinalis* have led to multiple outbreaks of diarrhea in both humans and bovine animals, causing great economic losses within the breeding industry and affecting human health [1]. Meanwhile, they are listed as pathogens that must be tested for in domestic water due to their high pathogenicity.

Knowledge about the distribution and composition of *Cryptosporidium* spp. and *G. intestinalis* helps with the recognition of the transmission of these pathogens. To date, more than 40 valid species have been reported in various animals [5]. Of these, over 20 species/genotypes are zoonotic, while others are usually identified in limited hosts [6]. Host adaption exists in the presence of *Cryptosporidium* spp., as reflected by the major occurrence of 1–4 species in one host [6]. Sequence analysis based on the triosephosphate isomerase (*tpi*), glutamate dehydrogenase (*gdh*), and β-giardin (*bg*) loci of *G. intestinalis* indicates there are eight genotypes with different host preferences, namely assemblage A-H [1,7,8]. Assemblages A and B can infect humans and many mammals. Assemblages C and D are usually recognized in canids, while assemblages E-H are usually identified in ruminants, felines, rodents, and aquatic animals, respectively [1,7,8].

Yaks are mainly distributed in the plateau region centered around the Qinghai–Tibet Plateau. Previous studies have reported the occurrence of *Cryptosporidium* spp. and *G. intestinalis* in yaks in Qinghai, Tibet, Gansu, and some other regions in China with positive rates of 1.4–30% [9,10,11,12] and 1.7–6% [10,12,13], respectively. Genetic diversity analysis indicated 12 species/genotypes (*C. parvum*, *C. canis*, *C. bovis*, *C. struthionis*, *C. ryanae*, *C. baileyi*, *C. andersoni*, *C. ubiquitum*, *C. hominis*, *C. xiaoi*, *C. suis*-like and *Cryptosporidium* new genotype) and three genotypes (assemblages A, B and E) for *Cryptosporidium* spp. and *G. intestinalis* in yaks, respectively [14,15,16]. Ganzi is one of the major important yak-breeding areas in Sichuan in China, located in the southeastern Tibetan plateau. As a common economic animal in Ganzi, yaks are kept in enclosures and have close contact with farmers, but the occurrence of *Cryptosporidium* spp. and *G. intestinalis* in yaks in this region was seldom explored. Considering the high pathogenicity of the two pathogens in yaks and the lack of information on their distribution and transmission in yaks in Ganzi, this study investigated the occurrence and genetic diversity of *Cryptosporidium* and *G. intestinalis* in yaks from three regions in Ganzi, China, which could enrich our understanding of the occurrence of these two pathogens and provide a foundation for understanding their transmission and zoonotic potential in yaks in Ganzi.

## 2. Materials and Methods

### 2.1. Sample Collection

**Figure 1:**
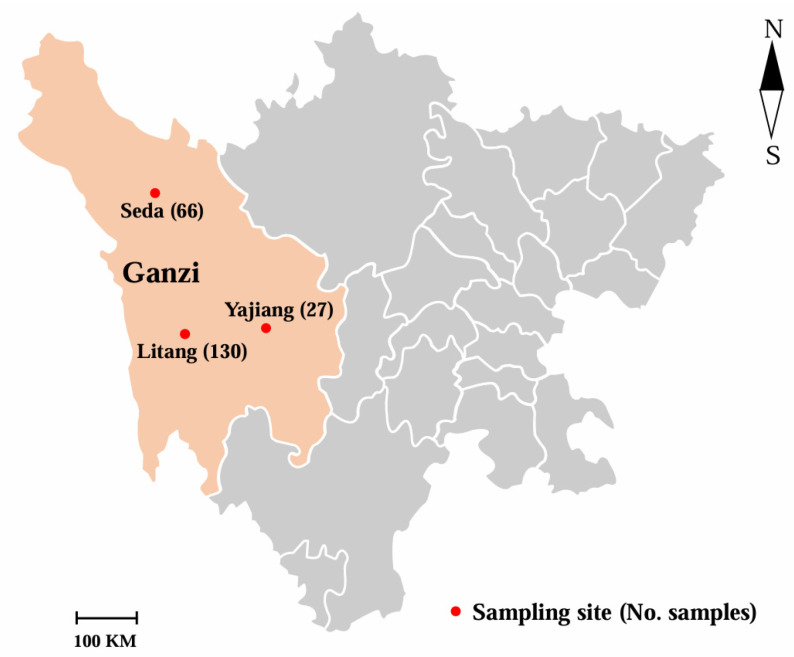
Distribution of three sampling sites (Seda, Yajiang, and Litang) of yak faeces in Ganzi. Red solid circles indicate sampling sites. The number in each bracket refers to the sample size.

Faecal specimens were sampled from 223 yaks kept in Seda, Yajiang, and Litang in Ganzi, China, as indicated in Figure 1. Seda, Yajiang, and Litang are the main production areas of yaks in Ganzi, and some yaks in these three regions experienced diarrhea according to the description of farmers. Those faecal specimens were randomly collected and accounted for 10.9% (66/603), 14.3% (27/189) and 10% (130/1296) of the total number of yaks in Seda, Yajiang, and Litang, respectively. Yaks in those investigated regions were housed, and did not mix with other livestock. Faecal specimens were directly and randomly collected from the rectum of yaks, and placed in disposable sampling bags marked with the yaks’ location, age, gender and diarrhea condition. Age groups were defined according to the growth and development condition of the yaks and verified by ear tags. The samples were then transmitted to the laboratory as soon as possible at a low temperature and preserved at −20 °C.

### 2.2. Genomic DNA Extraction

A commercial DNA isolation kit (Cate No. D4015-02; Omega Bio-Tek, Norcross, GA, USA) was used to isolate genomic DNA from 200 mg faecal specimens without pre-treatment as per the instructions. All the genomic DNA samples were preserved at −20 °C.

### 2.3. PCR Amplification

The occurrence and species of *Cryptosporidium* spp. were recognized by applying a nested PCR based on the *18S* rRNA gene with the size of ~830 bp [17]. The occurrence and genotypes of *G. intestinalis* were identified by using three nested PCRs targeting the *bg* [18], *gdh* [19], and *tpi* [20] loci with lengths of ~511 bp, ~392 bp and ~530 bp, respectively. The reaction system of PCRs (25 μL) contained 1 × Rapid *Taq* Master Mix (Cate No. P222-01, Vazyme, Nanjing, China), 0.4 μΜ of each primer (Table 1), 1 μL of gDNA for the primary PCR/1 μL primary PCR product for the secondary PCR under the PCR reaction conditions: denaturing occurred at 94 °C for 5 min, followed by 35 cycles of 94 °C for 45 s, annealing at the same temperature (Table 1) for 45 s, and 68 °C for 1 min, and an additional extension at 68 °C for 7 min. For each PCR, genomic samples of *Cryptosporidium xiaoi* and *G. intestinalis* assemblage C were used as positive controls for *Cryptosporidium* spp. and *G. intestinalis* PCR, respectively, and a negative control with ddH_2_O was also included. Positive secondary PCR products were confirmed by gel electrophoresis and then applied for sequencing at both directions using primers as PCRs.

### 2.4. Sequence Analysis

All the sequences were assemblaged, edited, and aligned by ChromasPro V1.5, BioEdit V7.0.5.3 and Clustal X V1.81, respectively. Phylogenetic trees for each locus were constructed using the Maximum Likelihood method with the bootstrap evaluation of 1000 replicates in MEGA V6.06.

### 2.5. Statistical Analysis

Differences in the occurrence of *Cryptosporidium* spp. and *G. intestinalis* among various factors were analyzed by applying an χ^2^ test within SPSS V22.0. Significant differences were recognized if the *p* value was less than 0.05. The OR (odds ratio) and RR (risk ratio) with 95% CIs (confidence intervals) were analyzed for the identification of factors associated with *Cryptosporidium* spp. and *G. intestinalis* infection.

### 2.6. Nucleotide Sequence Accession Numbers

Representative sequences for the *18S* rRNA, *bg*, *gdh*, and *tpi* loci found were submitted to GenBank^TM^ with the numbers PV151463-PV151469, PV157997-PV158007, PV297768-PV297771 and PV297772-PV297776, respectively.

## 3. Results

### 3.1. Occurrence of Cryptosporidium spp. in Yaks in Ganzi

The positive rate of *Cryptosporidium* spp. was 7.2% (16/223) in yaks in Ganzi. Among those three locations, the highest rate was found in yaks in Yajiang (37%, 10/27), followed by Seda (3%, 2/66) and Litang (3.1%, 4/130), and significant differences were found for the positive rates of *Cryptosporidium* spp. among the three locations (χ^2^ = 41.1316, df = 2, *p* < 0.0001). Meanwhile, significant differences were also recognized for the positive rates of *Cryptosporidium* spp. among yaks of different ages (χ^2^ = 14.7828, df = 3, *p* = 0.002), with the highest observed in yaks under 6 months of age (20.5%, 8/39), followed by yaks aged 6–12 months (7.8%, 4/51), >24 months (4.4%, 4/90), and 12–24 months (0, 0/43). Although a higher positive rate was found in male yaks and non-diarrheal yaks compared with female yaks and diarrheal yaks, respectively, no significant difference for the positive rate was found in relation to gender and diarrhea (Table 2).

**Figure 2:**
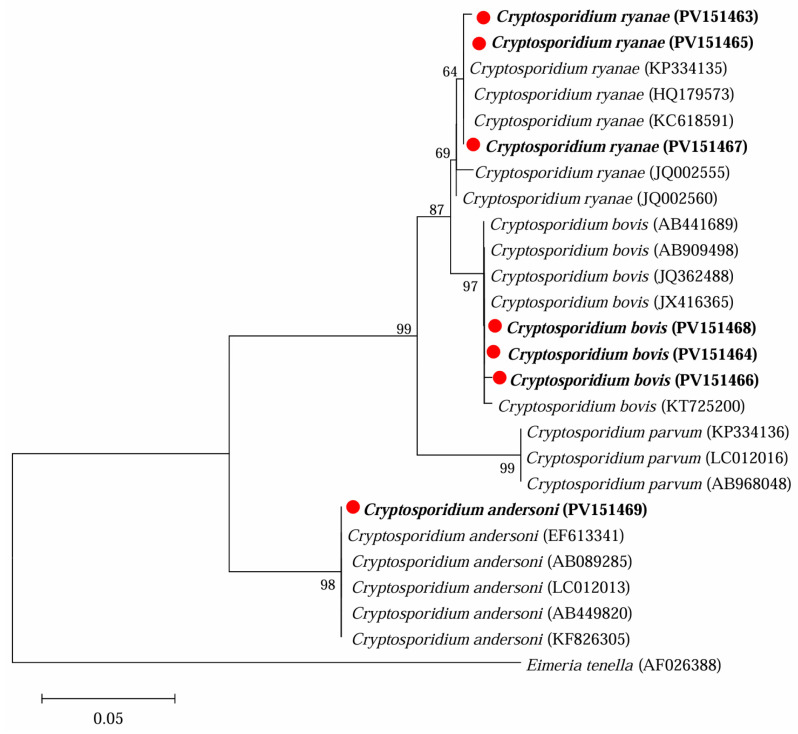
Phylogenetic analysis of representative sequences for the *18S* rRNA locus of *Cryptosporidium* species in this study with referenced sequences, obtained via Maximum Likelihood analysis. The sequences obtained in this study are marked with red solid circles and highlighted in bold font. *Eimeria tenella* (AF026388) was used as the outgroup; bootstrap values over 50% are indicated.

Sequence analysis of the *18S* rRNA locus identified three *Cryptosporidium* species in the 16 positive specimens, namely *C. bovis* (9), *C. ryanae* (6), and *C. andersoni* (1) (Table 2 and Figure 2). Among them, *C. bovis* was found in three locations and three age groups, *C. ryanae* in two locations and three age groups, while *C. andersoni* was found in only one location and one age group.

### 3.2. Occurrence of G. intestinalis in Yaks in Ganzi

The positive rate of *G. intestinalis* was 15.7% (35/223) in yaks in Ganzi, with 15.7% (35/223), 13.9% (31/223), and 7.2% (16/223) at *gdh*, *bg*, and *tpi* loci, respectively. Significant differences were found for the positive rates among three locations (χ^2^ = 23.2214, *df* = 2, *p* < 0.0001), with the highest in Yajiang (40.7%, 11/27), followed by Litang (17.7%, 23/130), and Seda (1.5%, 1/66).

The highest positive rate of *G. intestinalis* was found in yaks aged <6 months (35.9%, 14/39), followed by those aged 6–12 months (23.5%, 12/51), 12–24 months (11.6%, 5/43), and >24 months (4.4%, 4/90), and significant differences were found for the positive rates of *G. intestinalis* among the four age groups (χ^2^ = 23.5424, *df* = 3, *p* < 0.0001). However, there were no significant differences for *G. intestinalis* in terms of gender and diarrheal condition (Table 3).

**Figure 3:**
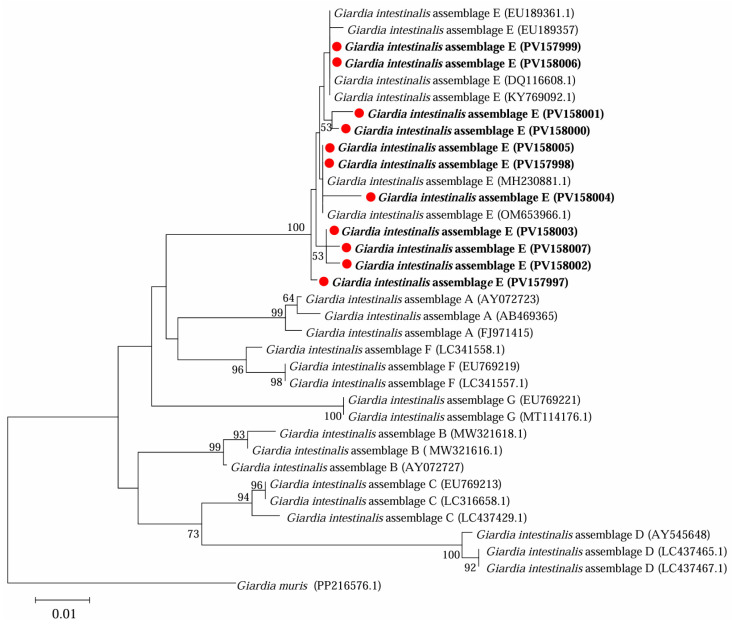
Phylogenetic analysis of representative sequences for the *bg* locus of *G. intestinalis* in this study with referenced sequences, obtained using Maximum Likelihood analysis. The sequences obtained in this study are marked with red solid circles and highlighted in bold font. *Giardia muris* (PP216576.1) was used as the outgroup; bootstrap values over 50% are indicated.

**Figure 4:**
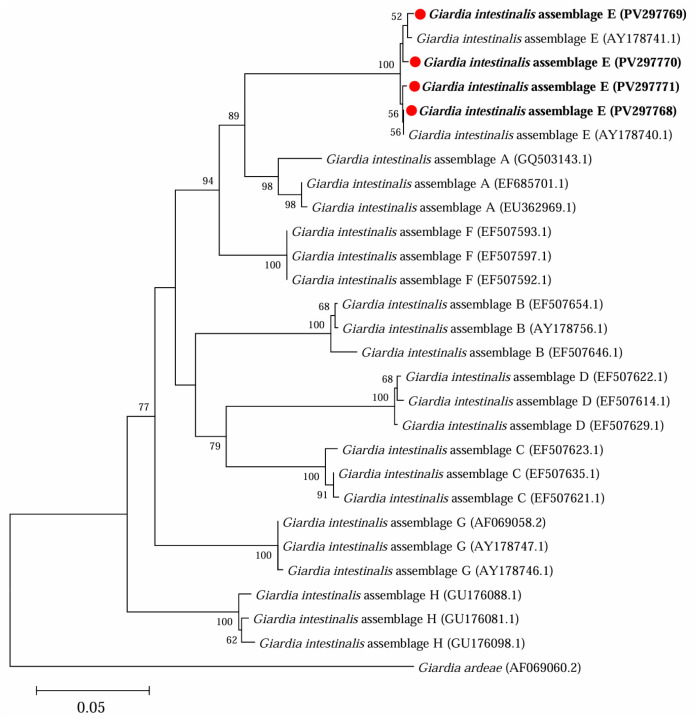
Phylogenetic analysis of representative sequences for the *gdh* locus of *G. intestinalis* in this study with referenced sequences obtained using Maximum Likelihood analysis. The sequences obtained in this study are marked with red solid circles and highlighted in bold font. *Giardia ardeae* (AF069060.2) was used as the outgroup; bootstrap values over 50% are indicated.

**Figure 5:**
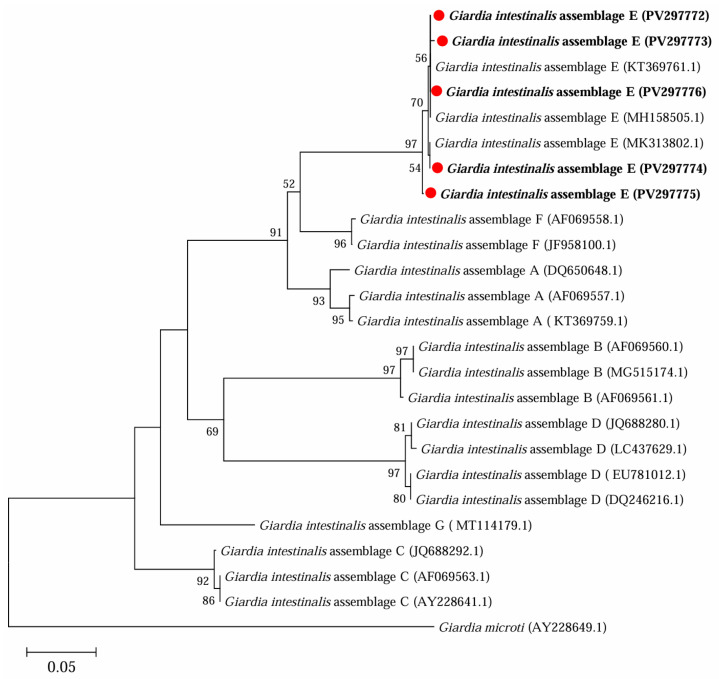
Phylogenetic analysis of representative sequences for the *tpi* locus of *G. intestinalis* in this study with referenced sequences obtained using Maximum Likelihood analysis. Those sequences obtained in this study are marked with red solid circles and highlighted in bold font. *Giardia microti* (AY228649.1) was used as the outgroup; bootstrap values over 50% are indicated.

Further phylogenetic analysis based on sequences of *bg* (Figure 3), *gdh* (Figure 4) and *tpi* (Figure 5) loci from the present study and referenced sequences downloaded from NCBI indicated that there existed one genotype (assemblage E) for *G. intestinalis* in yaks in the present study.

### 3.3. Co-Infection of Cryptosporidium spp. and G. intestinalis in Yaks in Ganzi

Notably, co-infection of *Cryptosporidium* spp. and *G. intestinalis* was found in five yaks. Among them, four yaks were co-infected with *C. ryanae* and *G. intestinalis*, and one with *C. bovis* and *G. intestinalis*.

## 4. Discussion

*Cryptosporidium* spp. and *G. intestinalis* are two common zoonotic protozoa that are closely related to diarrhea, threatening the health of humans and the breeding industry. This study applied nested PCR-based sequencing techniques to explore *Cryptosporidium* spp. and *G. intestinalis* infections in yaks in Ganzi, China, which could contribute to the understanding of the occurrence and zoonotic potential of those pathogens in yaks.

Several techniques have been applied in epidemiological studies of *Cryptosporidium* spp. and *G. intestinalis*. Traditional morphological methods are considered to be the “gold standard”, but these techniques are time-consuming, lack sensitivity under low infection intensity, and should be performed by an experienced person. PCR-based molecular methods are high-throughput and can make up for the deficiencies of traditional morphological methods to a certain extent [1], but can easily be polluted if the operation is not standardized. Therefore, it is necessary to follow a standard operation procedure and set suitable positive and negative controls in the PCR test.

The total positive rate of *Cryptosporidium* spp. in yaks was 7.2% (16/223) in Ganzi, which was higher than that in Tibet (1.4%, 8/577) [10], Gansu (5.26%, 4/76) [21], and the central western region of China (4%, 22/545) [12], but lower than that in Qinghai (30%, 98/327) [11]. Differences in the positive rates among regions were likely caused by several factors, such as geographic location, sample size, age, and animal condition. There existed significant differences related to the occurrence of *Cryptosporidium* spp. in yaks among different age groups, and the positive rate decreased with the increase in age. Similar results have also been reported in yaks in Qinghai [9,11], reflecting *Cryptosporidium* infection likely related to immunity state, and younger yaks were found to be more susceptible to *Cryptosporidium* compared with older ones. Considering the greater susceptibility of younger yaks to *Cryptosporidium* and potential transmission from older yaks to younger ones, it is better to provide separate pens according to age, avoid unnecessary contact with older yaks, and enhance daily management to reduce the possibility of infection.

Further sequence analysis highlighted three *Cryptosporidium* species (*C. bovis*, *C. andersoni* and *C. ryanae*), with *C. bovis* being the dominant one. Except for those three common species in bovine animals, several common zoonotic species (e.g., *C. parvum* and *C. canis*) [15] have also been reported in yaks, indicating yaks’ zoonotic potential for the transmission of *Cryptosporidium*. In this study, no zoonotic *Cryptosporidium* species were found, which was likely due to the differences in multifaceted factors, such as sampling size, climate, and management. Investigations on a wider scale are needed to comprehensively understand the composition and transmission of *Cryptosporidium* species. Considering the close contact between farmers and yaks in the investigated regions and the potential zoonotic transmission of *C. parvum*, *C. canis,* and other species, interventions based on WASH (water, sanitation, and hygiene) are needed to reduce the possibility of *Cryptosporidium* species being transmitted between humans and yaks.

The positive rate of *G. intestinalis* was 15.7% (35/223) in yaks in Ganzi, which was higher than that in Tibet (1.7%, 10/577) [10] and the central western region of China (6%, 16/545) [13]. Several factors, e.g., sampling size, age, and management, may have led to the differences in the occurrence of *G. intestinalis* in yaks among the regions. Meanwhile, significant differences in the positive rates of *G. intestinalis* in yaks among different age groups were identified, and the positive rate of *G. intestinalis* in yaks decreased with age. Considering the similarities in the age-related prevalence patterns of *Cryptosporidium* infection in yaks, similar interventions to those performed for *Cryptosporidium* are needed to reduce the possibility of *G. intestinalis* infections in younger animals with incomplete immunity.

Sequence analysis indicated that there was one genotype (assemblage E) of *G. intestinalis* in yaks in this study, which has also been identified in yaks in Tibet [10], Qinghai [14], and the central western region of China [12]. Assemblage E was commonly found in ruminants (e.g., dairy cattle, camels, sheep, and goats) [22,23,24,25]. In addition to assemblage E, zoonotic assemblages A and B have been found in yaks in Tibet [16] and Qinghai [14], respectively, indicating the possible zoonotic potential for the spread of *G. intestinalis* in yaks. The zoonotic potential for *G. intestinalis* in yaks in Ganzi needs to be further explored in more animals located in different geographic regions.

Notably, co-infection of *Cryptosporidium* spp. and *G. intestinalis* was observed in yaks. Previous studies have reported that mixed infection of *Cryptosporidium* spp. and *G. intestinalis* was also found in other ruminants, such as sheep and cattle [26,27,28]. Both *Cryptosporidium* spp. and *G. intestinalis* infection could lead to diarrhea and intestinal damage, which could provide convenient conditions and thus cause secondary infection of other intestinal pathogens, exacerbating intestinal damage. Moreover, previous studies found that co-infection with multiple intestinal pathogens likely caused more severe diarrhea compared with a single infection [29]. Thus, the relationship between co-infection of *Cryptosporidium* spp. and *G. intestinalis* and the severity of diarrhea and intestinal damage in yaks needs to be investigated in further studies to help improve our understanding of the pathogenesis of these two pathogens in yaks.

## 5. Conclusions

This study investigated the colonization of *Cryptosporidium* spp. and *G. intestinalis* in yaks from Ganzi, China, and *C. bovis*, *C. ryanae*, *C. andersoni* and *G. intestinalis* (assemblage E) identified. Significantly higher positive rates of *Cryptosporidium* spp. and *G. intestinalis* were found in yaks in Yajiang and yaks aged under 6 months, which indicated that location and age were likely to be risk factors. The results of the recent studies could enrich our knowledge about *Cryptosporidium* spp. and *G. intestinalis* infections in yaks in Ganzi, offering reference data which will be useful for understanding the transmission of those two pathogens in Ganzi and other related regions.

## Figures and Tables

**Table 1:** Sequence information of the primers used for PCRs in this study.

Targets	Primer Names	Sequence (5′-3′)	Annealing Temperature
*18S* rRNA [17]	*18S*-F1	TTCTAGAGCTAATACATGCG	55 °C
	*18S*-R1	CCCATTTCCTTCGAAACAGGA	
	*18S*-F2	GGAAGGGTTGTATTTATTAGATAAAG	55 °C
	*18S*-R2	CTCATAAGGTGCTGAAGGAGTA	
*bg* [18]	*bg*-F1	AAGCCCGACGACCTCACCCGCAGTGC	65 °C
	*bg*-R1	GAGGCCGCCCTGGATCTTCGAGACGAC	
	*bg*-F2	GAACGAGATCGAGGTCCG	55 °C
	*bg*-R2	CTCGACGAGCTTCGTGTT	
*gdh* [19]	*gdh*-F1	TTCCGTGTCCAGTACAACTC	50 °C
	*gdh*-R1	GCCAGCTTCTCCTCGTTGAA	
	*gdh*-F2	CGCTTCCACCCCTCTGTCAAT	50 °C
	*gdh*-R2	TGTTGTCCTTGCACATCTC	
*tpi* [20]	*tpi*-F1	AATAAATIATGCCTGCTCGTCG	54 °C
	*tpi*-R1	ATGGACITCCTCTGCCTGCTC	
	*tpi*-F2	CCCTTCATCGGIGGTAACTTCAA	58 °C
	*tpi*-R2	GTGGCCACCACICCCGTGCC	

**Table 2:** Occurrence of *Cryptosporidium* spp. in yaks in Ganzi, Sichuan Province.

Factors	No. Tested	No. Positive (%, 95% CI)	RR (95% CI)	OR (95% CI)	*p*	Species (n)
**Location**						
Yajiang	27	10 (37.0, 0.2153–0.5577)	0.0831 (0.0281–0.2454)	0.054 (0.0152–0.1913)	<0.0001	*C. bovis* (7), *C. ryanae* (3)
Seda	66	2 (3.0, 0.0083–0.1039)	1.0154 (0.1909–5.4012)	1.0159 (0.1812–5.6951)		*C. bovis* (1), *C. andersoni* (1)
Litang	130	4 (3.1, 0.012–0.0765)	Reference	Reference		*C. ryanae* (3), *C. bovis* (1)
**Age (Months**)						
<6	39	8 (20.5, 0.1078–0.3553)	0.2167 (0.0693–0.6774)	0.1802 (0.0507–0.6408)	0.002	*C. bovis* (5), *C. ryanae* (3)
6–12	51	4 (7.8, 0.0309–0.185)	0.5667 (0.148–2.1699)	0.5465 (0.1307–2.2855)		*C. bovis* (3), *C. ryanae* (1)
12–24	43	0 (0, 0–0.082)	-	-		-
>24	90	4 (4.4, 0.0174–0.1087)	Reference	Reference		*C. ryanae* (2), *C. bovis* (1), *C. andersoni* (1)
**Gender**						
Male	62	3 (4.8, 0.0166–0.1329)	0.3444 (0.0368–3.22)	0.3333 (0.0337–3.2976)	0.3253	*C. ryanae* (2), *C. bovis* (1)
Female	60	1 (1.7, 0.003–0.0886)	Reference	Reference		*C. ryanae* (1)
NA	101	12 (11.9, 0.0693–0.1963)				*C. bovis* (8), *C. ryanae* (3), *C. andersoni* (1)
**Diarrhea**						
Yes	40	3 (5.0, 0.0258–0.1986)	1.0558 (0.3155–3.5331)	1.0603 (0.2876–3.9092)	0.9299	*C. bovis* (1), *C. ryanae* (1), *C. andersoni* (1)
No	183	13 (7.1, 0.042–0.1177)	Reference	Reference		*C. bovis* (8), *C. ryanae* (5)
**Total**	223	16 (7.2, 0.0446–0.1133)	-	-		*C. bovis* (9), *C. ryanae* (6), *C. andersoni* (1)

NA: not available; CI: confidence interval; RR: risk ratio; OR: odds ratio; Reference: reference group for pairwise comparison.

**Table 3:** Occurrence and risk factors of *G. intestinalis* infection in yaks in Ganzi, Sichuan Province.

Factors	No. Tested	No. Positive (%, 95% CI)			RR (95% CI)	OR (95% CI)	*p*	Genotype (n)		
		*gdh*	*tpi*	*bg*				*gdh*	*tpi*	*bg*
**Location**										
Yajiang	27	11 (40.7, 0.2451–0.5927)	4 (14.8, 0.0591–0.3247)	10 (37, 0.2153–0.5577)	0.4343 (0.2415–0.781)	0.3127 (0.1284–0.7614)	<0.0001	E (11)	E (4)	E (10)
Seda	66	1 (1.5, 0.0027–0.081)	0 (0, 0–0.055)	0 (0, 0–0.055)	11.6769 (1.612–84.583)	13.972 (1.8428–105.9317)		E (1)	-	-
Litang	130	23 (17.7, 0.1209–0.2515)	12 (9.2, 0.0536–0.1544)	21 (16.2, 0.1081–0.2343)	Reference	Reference		E (23)	E (12)	E (21)
**Age (Months)**										
<6	39	14 (35.9, 0.2274–0.5158)	8 (20.5, 0.1078–0.3553)	13 (33.3, 0.2063–0.4902)	0.1238 (0.0435–0.3523)	0.0831 (0.0251–0.275)	<0.0001	E (14)	E (8)	E (13)
6–12	51	12 (23.5, 0.1401–0.3676)	5 (9.8, 0.0426–0.2097)	12 (23.5, 0.1401–0.3676)	0.1889 (0.0643–0.5552)	0.1512 (0.0458–0.4985)		E (12)	E (5)	E (12)
12–24	43	5 (11.6, 0.0507–0.2448)	2 (4.7, 0.0128–0.1545)	4 (9.3, 0.0368–0.216)	0.3822 (0.108–1.3524)	0.3535 (0.0899–1.3899)		E (5)	E (2)	E (4)
>24	90	4 (4.4, 0.0174–0.1087)	1 (1.1, 0.002–0.0603)	2 (2.2, 0.0061–0.0774)	Reference	Reference		E (4)	E (1)	E (2)
**Gender**										
Male	62	10 (16.1, 0.09–0.2721)	4 (6.5, 0.0254–0.1545)	10 (16.1, 0.09–0.2721)	1.1367 (0.5214–2.478)	1.1673 (0.4555–2.9917)	0.7471	E (10)	E (4)	E (10)
Female	60	11 (18.3, 0.1056–0.2992)	3 (5, 0.0171–0.137)	9 (15, 0.081–0.2611)	Reference	Reference		E (11)	E (3)	E (9)
NA	101	14 (13.9, 0.0844–0.2193)	9 (8.9, 0.0476–0.1607)	12 (11.9, 0.0693–0.1963)	-	-		E (14)	E (9)	E (12)
**Diarrhea**										
Yes	40	6 (15.0, 0.0706–0.2907)	2 (5, 0.0138–0.165)	2 (5, 0.0138–0.165)	1.0565 (0.4701–2.3743)	1.0671 (0.4109–2.7711)	0.8939	E (6)	E (2)	E (2)
No	183	29 (15.9, 0.1127–0.2184)	14 (7.7, 0.0461–0.1243)	29 (15.9, 0.1127–0.2184)	Reference	Reference		E (29)	E (14)	E (29)
**Total**	223	35 (15.7, 0.1151–0.2105)	16 (7.2, 0.0446–0.1133)	31 (13.9, 0.0997–0.1905)	-	-		E (35)	E (16)	E (31)

NA: not available; CI: confidence interval; RR: risk ratio; OR: odds ratio; Reference: reference group for pairwise comparison.

## Data Availability

Data is contained within the article.
